# Extraskeletal Ewing Sarcoma Mimicking Benign Soft Tissue Tumors: A Rare Case

**DOI:** 10.7759/cureus.98009

**Published:** 2025-11-28

**Authors:** Mohammed A Aldabbas, Mohammad M Alshorman, Laith N Albudour, Zamel M Al Fohili, Tasneem N Al-Malahmeh

**Affiliations:** 1 Department of Plastic and Reconstructive Surgery, Jordanian Royal Medical Services, Amman, JOR; 2 Department of Histopathology, Jordanian Royal Medical Services, Amman, JOR

**Keywords:** cd99, ckit, cyclin d1, extraskeletal ewing sarcoma, vimentin

## Abstract

Extraskeletal Ewing sarcoma (EES) is a rare subtype within the Ewing sarcoma family of tumors (ESFT), typically presenting as a large soft tissue mass. Imaging features of EES are generally non-specific and vary depending on the tumor’s location and the tissues involved. Magnetic resonance imaging (MRI) plays a crucial role in the initial evaluation, local staging, and ongoing management. A definitive diagnosis requires core needle biopsy, immunochemistry, and histopathological confirmation. Treatment typically involves systemic chemotherapy combined with surgical excision when feasible. Due to the tumor’s tendency for large size and local invasion, a multidisciplinary approach is essential for optimal management.

A 23-year-old female patient with no significant medical and surgical history presented with a complaint of a slowly enlarging mass over the lateral right thigh that has been growing for an estimated duration of three years, according to the patient. The mass measured approximately 2 × 2 to 3 × 4 cm and was firm, round, non-tender, and associated with occasional episodes of bloody discharge. Ultrasound (US) imaging showed a hypoechoic, oval, subcutaneous lesion measuring 1 × 1.4 cm with regular margins. Based on clinical and imaging findings, differential diagnoses included hemangioma, angiolipoma, leiomyoma, neurolipoma, and dermatofibroma. The patient underwent surgical excision of the lesion through an elliptical incision. Gross examination revealed a 6 × 4 cm skin ellipse with an underlying 7 × 4.5 × 2.5 cm mass, appearing whitish and lobulated, with a focal ulceration. Histopathological analysis confirmed the diagnosis of EES, grade 3, with a mitotic rate of 25/10 HPF. Immunohistochemistry demonstrated strong diffuse CD99 positivity along with positivity for vimentin, BCL2, Cyclin D1, and C-Kit, with a high Ki-67 proliferation index (85%-90%). No lymphovascular invasion, nodal involvement, or distant metastasis was detected, and staging was pT1.

Following multidisciplinary discussion, the patient was initiated on adjuvant chemotherapy using the CAV-IE (cyclophosphamide, doxorubicin, vincristine alternating with ifosfamide and etoposide), with a plan to complete 12 cycles, while radiotherapy was not indicated. She commenced her treatment with Cycle 1 of the IE component, accompanied by supportive care including intravenous fluids, ondansetron, omeprazole, and dexamethasone. As of the latest update, the patient remains admitted and is currently on Day 4 of her first IE cycle. She developed dysuria during hospitalization, and the treatment plan was adjusted to include an increased dose of mesna to enhance uroprotection. This case highlights the importance of considering EES in the differential diagnosis of persistent soft tissue masses, even in slow-growing lesions. It underscores the pivotal role of immunohistochemistry and multidisciplinary management in achieving a definitive diagnosis and favorable outcomes.

## Introduction

Extraskeletal Ewing sarcoma (EES) is a rare and rapidly growing malignant small round-cell tumor (SRCT) that arises in the soft tissues and can develop at almost any anatomical site. It predominantly affects adolescents and young adults between 10 and 30 years of age and is characterized by an aggressive clinical course and high mortality rate [1,2]. Because of this aggressive behavior, EES often presents late, typically with soft tissue swelling, pain, or neurological symptoms caused by compression of adjacent nerves [3,4]. EES continues to be an uncommon and diagnostically troublesome cancer with extremely variable anatomical presentations, which tend to masquerade as other soft tissue tumors and provoke delayed or incorrect diagnosis [5].

Accurate diagnosis relies on a combination of histological, immunohistochemical, and cytogenetic assessments. Immunophenotyping, a technique used to identify cell-surface and intracellular markers, is central to the diagnostic process. Most EES tumors show strong CD99 (MIC2) positivity; however, CD99 is not entirely specific, so additional markers such as NKX2.2 are used to differentiate EES from other SRCTs. Cytogenetic analysis provides further confirmation. More than 90% of cases demonstrate the characteristic t(11;22)(q24;q12) chromosomal translocation, which produces the EWS-FLI1 fusion transcript-a genetic hallmark of Ewing sarcoma family tumors. Despite its diagnostic value, genetic testing may be limited by access to specialized assay kits [1,6-8].

Imaging findings are often non-specific, but modalities such as MRI, CT, Doppler ultrasound, and positron emission tomography (PET)/CT help evaluate the extent of disease, involvement of surrounding structures, and possible metastases. Emerging tools such as liquid biopsy also show promise as non-invasive methods for diagnosis and disease monitoring [2,9].

Treatment approaches for EES generally mirror those used for osseous Ewing sarcoma. Management typically involves multimodal therapy with surgical resection, systemic chemotherapy, and radiotherapy. Despite these strategies, the prognosis remains poor, particularly in patients with large tumors, metastases at presentation, or incomplete surgical margins. Reported two-year survival rates are below 40%, and some studies describe survival dropping to less than 10% at three years. Early diagnosis, aggressive multimodality treatment, and advancements in molecular diagnostics are, therefore, critical to improving outcomes [10-13].

We hypothesize that EES should be considered in the differential diagnosis of persistent soft tissue masses, even when lesions appear slow-growing. This case highlights the essential role of immunohistochemistry (IHC) and multidisciplinary management in achieving a definitive diagnosis and formulating an optimal therapeutic plan.

## Case presentation

A 23-year-old patient with no significant medical and surgical history or known drug allergies presented to the surgical outpatient clinic with a complaint of a mass in her right thigh. She reported having this lesion for over 2-3 years, but she noticed it had been gradually increasing in size over the past 2-3 months, with periods of regression and reappearance, according to the patient’s observation. Initially, there was no associated pain or discharge, but over time, the mass increased in size and was later complicated by intermittent episodes of bloody discharge.

**Figure 1:**
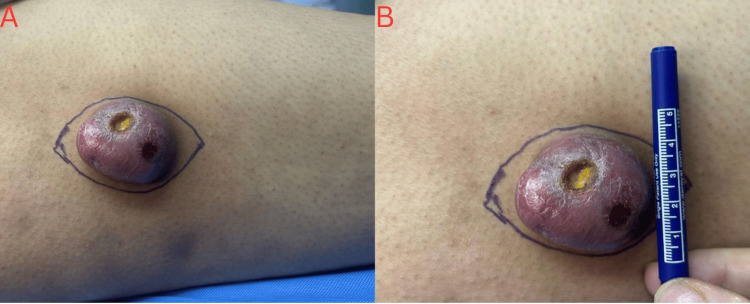
Preoperative appearance of the right lateral thigh mass. (A) Ulcerated, erythematous, and necrotic subcutaneous lesion with clearly demarcated margins, consistent with an aggressive soft tissue process. (B) Measurement demonstrating a maximal diameter of approximately 5 cm

On physical examination, the patient appeared well, afebrile, and vitally stable. Local inspection revealed a palpable, round, non-tender, regular subcutaneous mass over the lateral aspect of the right thigh, measuring approximately 2 × 2 cm without overlying skin changes. As the lesion progressed, it increased to approximately 3 × 4 cm. The patient was referred to dermatology, where differential diagnoses included angiolipoma, leiomyoma, dermatofibroma, and neurofibroma. Given the rapid enlargement and ulceration of the lesion (Figure 1), surgical excision was recommended.

**Figure 2:**
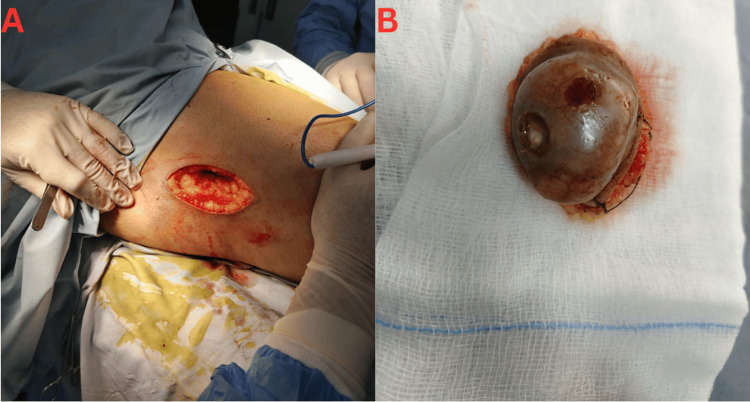
Intraoperative findings and gross specimen. (A) Wide local excision of the right thigh mass, showing involvement of subcutaneous tissue without deep fascial infiltration. (B) Resected specimen demonstrating a well-circumscribed, necrotic, ulcerated mass consistent with a high-grade malignant tumor

On March 6, 2025, the patient underwent a wide local excision of the lesion. Intraoperatively, the lesion appeared lobulated with surface ulceration and focal hemorrhage (Figure 2A). The resected specimen demonstrated a well-circumscribed necrotic mass (Figure 2B). Grossly, the excised specimen measured 4.5 × 3.3 × 3.4 cm. Postoperative recovery was uneventful. Histopathological examination confirmed a malignant round blue cell tumor with immunomorphological features most consistent with EES. The tumor was staged as pT1 according to the American Joint Committee on Cancer (AJCC) 8th edition [14]. The mitotic index was high (25 mitoses per 10 high-power fields), and necrosis was present in approximately 5% of the tumor. The closest margin was 0.4 cm laterally, and no lymph nodes were submitted. All surgical margins were negative.

**Figure 3:**
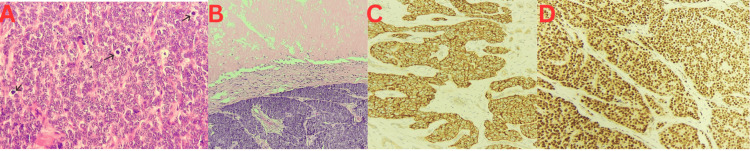
Histopathological and immunohistochemical (IHC) features of the excised tumor. (A) H&E, 400×: sheets of small round blue cells with scant cytoplasm, hyperchromatic nuclei, prominent nucleoli, and frequent mitoses (arrows), characteristic of Ewing sarcoma family tumors. (B) H&E, 200×: tumor nests with focal necrosis. (C) CD99 IHC: diffuse strong membranous positivity, supporting Ewing sarcoma. (D) FLI-1 IHC: strong nuclear expression, further supporting Ewing sarcoma lineage

IHC revealed diffuse strong positivity for CD99, as well as positivity for vimentin, BCL2, Cyclin D1, and C-Kit. INI-1 showed retained nuclear expression, and importantly, FLI-1 demonstrated strong diffuse nuclear positivity. Other markers, including CK5/6, CK20, CK7, EMA, BerEP4, Synaptophysin, CD56, NSE, CD79a, LCA, CK8/18, Desmin, S100, CD34, TLE-1, CEA, SMA, Calretinin, WT-1, Inhibin, HMB45, CD68, CD21, and SATB2, were all negative. The Ki-67 proliferative index was estimated at 85%-90%, indicating a high-grade tumor. Molecular studies including EWSR1-FLI1 translocation testing were recommended for confirmatory diagnosis. Histopathology showed classic features of a malignant SRCT with high mitotic activity and focal necrosis (Figures 3A, 3B). Immunohistochemical staining demonstrated diffuse CD99 and nuclear FLI-1 positivity (Figures 3C, 3D), strongly supporting a diagnosis of EES.

**Figure 4:**
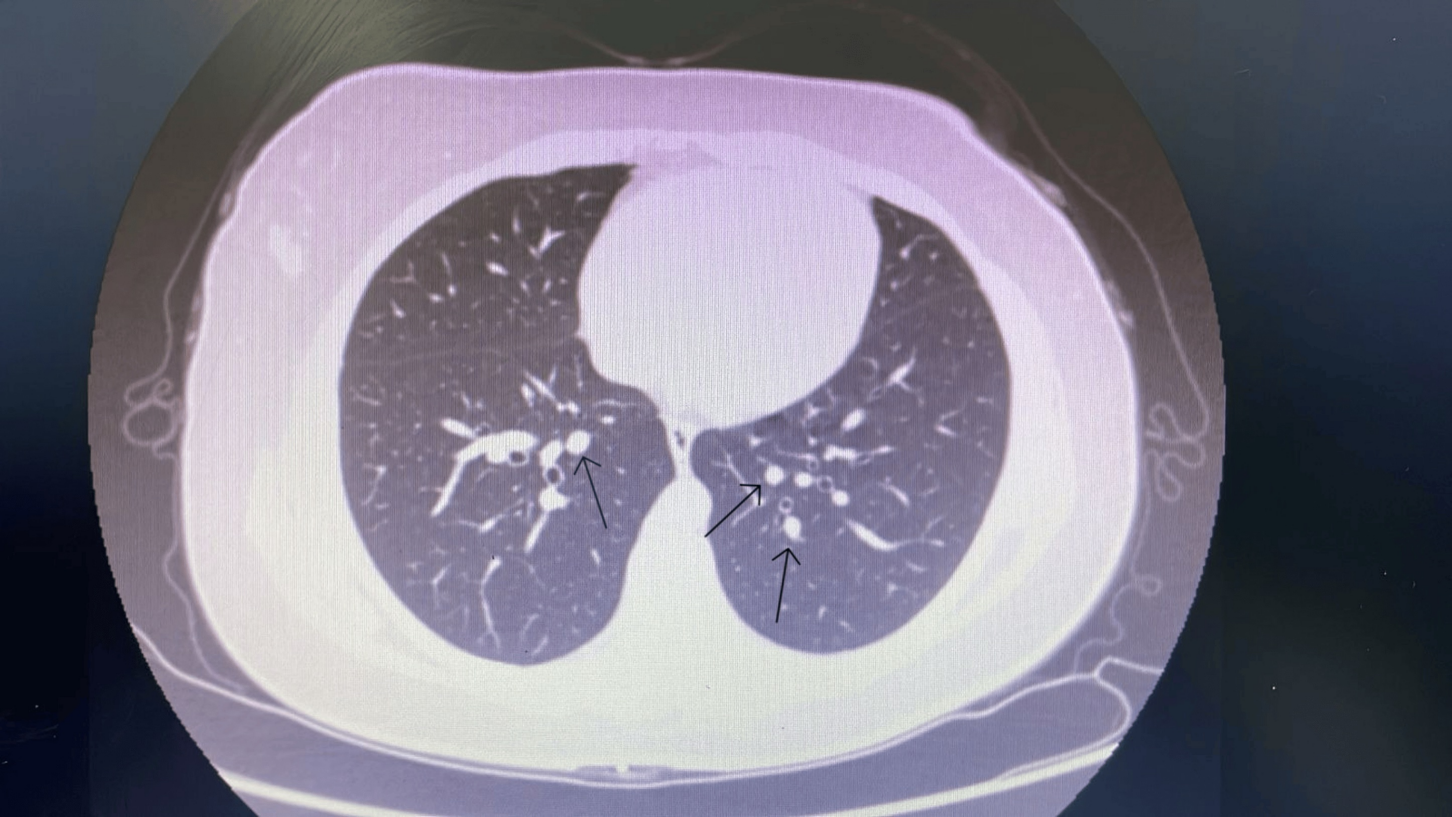
Axial contrast-enhanced CT of the chest demonstrating two subcentimeter pulmonary nodules. A 3.2 mm nodule is seen in the left lower lobe (black arrow), and a 3 mm subpleural nodule is noted in the right middle lobe (black arrow). These indeterminate nodules were recommended for interval surveillance given the patient’s new diagnosis of a high-grade small round-cell tumor

Following excision, the patient underwent comprehensive staging investigations. MRI of the lower extremity demonstrated no evidence of residual tumor, soft tissue recurrence, or bony involvement, with only postoperative subcutaneous changes noted. Contrast-enhanced CT of the chest revealed two small indeterminate pulmonary nodules: a 3.2 mm left lower lobe nodule and a 3 mm subpleural right middle lobe nodule, both of which were recommended for interval surveillance (Figure 4). No focal liver lesions or lymphadenopathy were identified. A small thyroid lesion was also incidentally detected, for which a dedicated thyroid ultrasound was advised. A whole-body bone scan showed no evidence of skeletal metastases.

Initial laboratory evaluation revealed severe microcytic anemia, with hemoglobin of 4.9 g/dL (Table 1), likely secondary to chronic tumor-related blood loss, and a mean corpuscular volume (MCV) of 39.8 fL, consistent with microcytosis. There were no signs of leukocytosis or thrombocytosis with normal white blood cell count (4,000-11,000/µL) and platelet count (150,000-400,000/µL), and liver function tests were within normal limits: aspartate aminotransferase (AST) (10-40 U/L), alanine transaminase (ALT) (7-56 U/L), alkaline phosphatase (ALP) (44-147 U/L), total bilirubin (0.1-1.2 mg/dL), and albumin (3.5-5.0 g/dL) (Table 1).

**Table 1:** Baseline laboratory findings with corresponding reference ranges

Test type	Result	Reference range
Hemoglobin level	4.9 g/dL	12.0-15.5 g/dL for females; 13.5-17.5 g/dL for males
Hematocrit	13.8%	36%-46% for females; 41%-53% for males
Mean corpuscular volume (MCV)	39.8 fL	80-100 fL

Subsequent laboratory follow-up showed an improvement in hemoglobin to 11.4 g/dL and hematocrit to 33.2%, consistent with mild normocytic anemia. White blood cell and platelet counts remained within normal ranges.

The case was reviewed in a multidisciplinary tumor board meeting. Given the complete excision with clear margins, radiotherapy was not indicated. The patient was referred to medical oncology for systemic treatment and genetic confirmation. Based on the tumor board’s recommendation, the patient was planned for 12 cycles of adjuvant chemotherapy using the alternating CAV-IE protocol (cyclophosphamide, doxorubicin, vincristine alternating with ifosfamide and etoposide).

Chemotherapy was initiated on May 1, 2025, beginning with the IE regimen. On Day 5 of Cycle 1, the patient received etoposide at 100 mg/m² as a one-hour intravenous (IV) infusion (total 176 mg) and ifosfamide at 1,800 mg/m² via an eight-hour infusion (total 3,168 mg). To prevent hemorrhagic cystitis, mesna was administered at 1,800 mg/m², corresponding to 100% of the ifosfamide dose, for a total of 7,128 mg given over eight hours. Premedication included ondansetron 8 mg IV. The patient also received IV hydration with 2 L of normal saline over 24 hours, along with Omedar 20 mg daily for gastric protection. On Day 4 of the cycle, the patient experienced mild dysuria, leading to an increase in the mesna dose, which resolved the issue.

**Figure 5:**
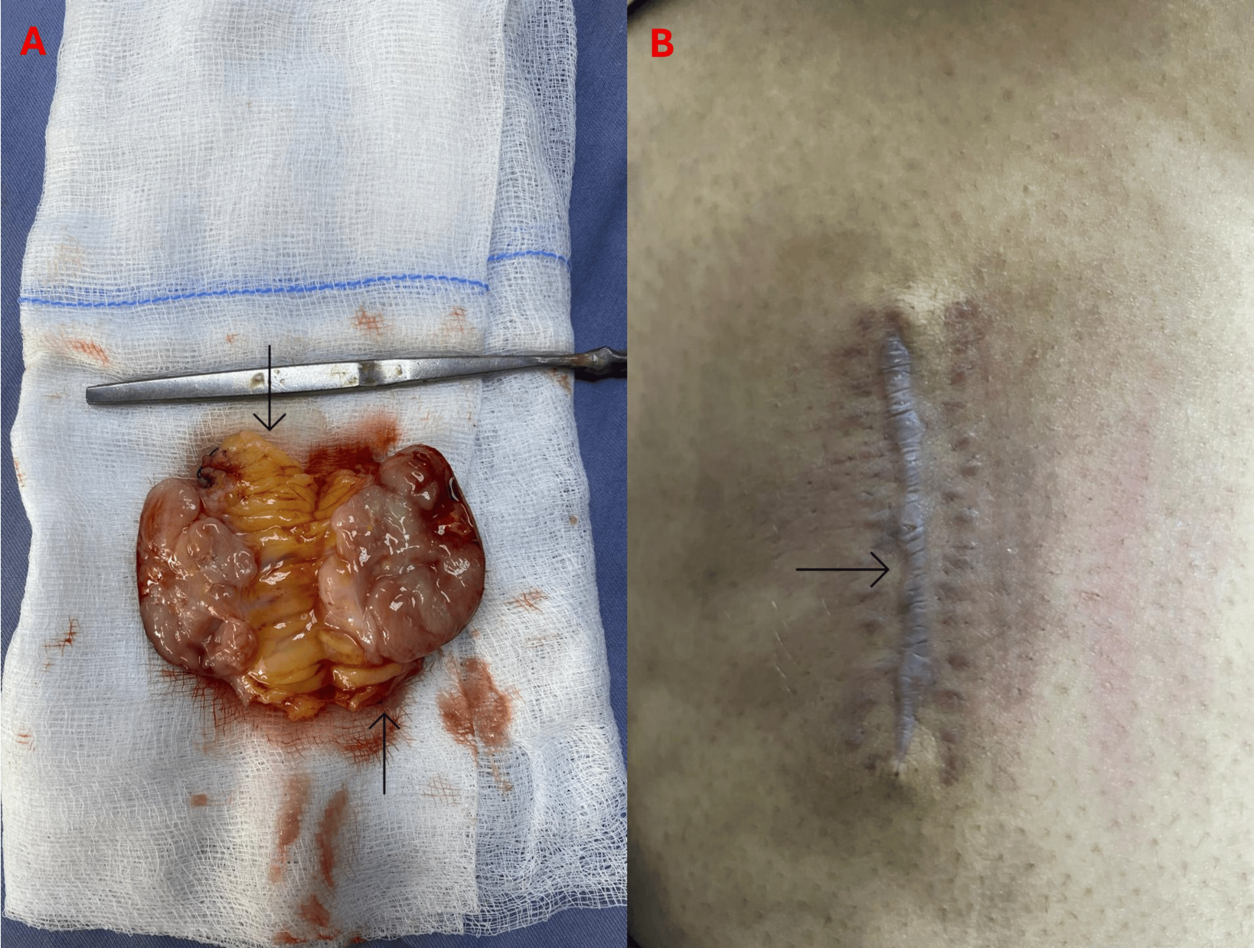
Postoperative findings following wide local excision of the right thigh mass. (A) Gross specimen showing a well-circumscribed, lobulated soft tissue tumor with focal necrosis (arrows) and adjacent fatty tissue. (B) Healed lateral thigh incision at follow-up demonstrating satisfactory wound closure with no signs of infection or dehiscence (arrow)

The patient subsequently completed Cycle 2 (VAC regimen: vincristine 2 mg IV, doxorubicin 75 mg/m², and cyclophosphamide 1,200 mg/m²) and Cycle 3 (IE), both of which were well tolerated without significant complications. At follow-up, the surgical site demonstrated satisfactory healing with no signs of infection or wound-related issues, and the gross postoperative specimen showed a well-circumscribed lobulated mass with focal necrosis, consistent with a high-grade SRCT (Figure 5). She remains under regular oncology surveillance with no systemic symptoms or clinical evidence of recurrence after three months. The multidisciplinary team (MDT) continues to monitor her response to treatment and awaits molecular confirmation of the EWSR1-FLI1 translocation to finalize staging and guide long-term follow-up.

## Discussion

In this report, we present a case of a 23-year-old female patient with no significant past medical or surgical history who developed a right thigh mass, later diagnosed as EES. This finding is consistent with previously published data indicating that EES predominantly affects individuals between 10 and 30 years of age, with no strong gender predilection overall [15]. However, some studies have noted a slight female predominance among older patients, which supports our case's demographics [16]. Moreover, it has been shown that patients with EES present at a higher median age (25 years) compared to those with skeletal Ewing sarcoma (16 years), a difference that is statistically significant [17,18]. This age pattern further corroborates the typical presentation seen in our patient.

Our patient presented with a right thigh mass that initially was small and non-tender but grew over time and developed bloody discharge. This presentation partially aligns with previous reports describing primary cutaneous Ewing sarcoma as small, slow-growing, soft, and occasionally hemorrhagic lesions on the extremities of young adults [15]. However, compared to the typically small and localized tumors reported, our case showed more rapid enlargement and signs of discharge, suggesting a more aggressive behavior. Imaging findings in the literature, such as large heterogeneous masses with internal necrosis or hemorrhage, are consistent with the clinical progression seen in our patient [19]. The literature supports this as well, as a similar case in the literature involves a 13-year-old girl who presents post-trauma with arm swelling. The lesion is diagnosed as a hematoma, but after one year of growth and a negative vascular ultrasound, the hematoma is re-excised and Ewing sarcoma is found on MRI/biopsy. Our patient's hematoma is excised and, per imaging, reconsidered after noted growth, only to find the same thing as in this report. They present in a similar way as benign, more commonly seen processes, but ultimately are much worse. But by then, the damage had been done. However, the patient had post-trauma growth, and she had delayed surgical excision, whereas our patient had an earlier surgical excision with earlier diagnosis and treatment [19].

In our case, initial ultrasound imaging of the right thigh revealed a small, well-circumscribed hypoechoic lesion with central color flow, suggesting a benign vascular lesion such as a hemangioma. This reflects the non-specific nature of imaging findings in EES, as highlighted in previous studies [15]. While imaging modalities such as ultrasound, CT, and MRI are crucial for assessing tumor size, vascular involvement, necrosis, and staging, they rarely provide a definitive diagnosis for EES. MRI findings typically include heterogeneous signal intensities with features of hemorrhage, necrosis, and soft tissue invasion, whereas CT can better define tumor margins and identify adjacent structural involvement [19]. However, as supported by the literature, no imaging pattern is pathognomonic for EES, and radiologic findings often overlap with other soft tissue tumors or vascular anomalies [15]. A PET scan is a crucial diagnostic tool for Ewing sarcoma; it has proven its reliability for staging, detecting metastases, and monitoring treatment response. Additionally, a PET scan has been shown to be helpful in accurately locating primary sarcoma and its margins. The key feature of this imaging technique is the option to record lesion progression or regression and detect chances of recurrence [20]. In our patient, despite imaging suggesting a benign process, the clinical progression to a larger mass with bloody discharge prompted surgical excision and further pathological evaluation.

Definitive diagnosis was achieved through histopathological and immunohistochemical evaluation of the excised specimen. Microscopically, the tumor displayed the characteristic features of EES: small, monomorphic round blue cells located within the dermis and subcutaneous tissue, a high mitotic rate, and focal tumor necrosis [21]. Immunohistochemical analysis showed strong diffuse positivity for CD99, alongside vimentin, BCL2, Cyclin D1, and C-Kit expression, while markers such as CK7, CK20, S100, and CD34 were negative, effectively ruling out differential diagnoses such as rhabdomyosarcoma, lymphoma, and other SRCTs [21,22]. This diagnostic approach is consistent with existing literature, which emphasizes that while CD99 is highly sensitive for Ewing sarcoma family tumors, it lacks specificity [15], making molecular confirmation-such as identification of the EWSR1-FLI1 translocation-critical. Our findings reaffirm that despite advances in imaging techniques, the diagnosis of EES relies predominantly on histopathological features and immunohistochemical profiling, supplemented by molecular testing when necessary.

In this case, another strategy can be proposed. Confirm diagnosis by fine needle biopsy initially, then start with neoadjuvant chemotherapy then undergo resection. This strategy helps to make a complete surgical resection [15,16,22]. In our case, following an MDT discussion, the patient was scheduled for 12 cycles of adjuvant CAV-IE chemotherapy with no indication for radiotherapy. The first cycle of the IE regimen was initiated, supported with IV hydration and Omedar 20 mg once daily. Given the absence of metastasis and the successful achievement of negative margins, our case supports the current understanding that surgery with wide, clear margins remains the primary and most effective treatment for localized EES, although the final treatment plan should be guided by molecular findings and multidisciplinary evaluation. One study shows that a multidisciplinary approach consisting of surgical resection and radiotherapy plus chemotherapy has increased the five-year survival rate from 20% to 58% [23,24].

Notably, patients with EES tend to have a higher incidence of distant metastasis than skeletal ES patients; it has been reported that 30%-40% of patients had distant metastasis disease at the time of diagnosis [21]. In our case, the patient did not have metastatic lesions and was treated successfully by surgical excision of the lesion through an elliptical incision. Postoperative MRI confirmed the absence of residual disease. Blood investigations, including a complete blood count, were within normal limits.

One of the key strengths of our case report is the detailed documentation of the patient’s clinical progression, imaging findings, surgical intervention, and histopathological analysis. This comprehensive approach highlights the challenges of diagnosing EES, especially when initial imaging suggested a benign lesion. Furthermore, the report reinforces the importance of surgical excision with wide margins for localized disease and contributes to the limited literature describing cutaneous or subcutaneous EES presentations in young adults.

However, the report also has limitations. Molecular confirmation through the detection of the EWSR1-FLI1 translocation, although recommended, was pending at the time of writing, which limits the completeness of the diagnostic workup. Moreover, the short follow-up period prevents assessment of long-term outcomes, recurrence rates, or the eventual need for adjuvant therapies. Finally, as a single case report, our findings cannot be generalized to all patients with EES, given the variability in clinical behavior and response to therapy.

## Conclusions

This case highlights the diagnostic challenges of EES and emphasizes the importance of maintaining a broad differential diagnosis when evaluating rapidly evolving soft tissue masses in young adults. Definitive diagnosis in our patient was achieved through histopathology and IHC, with complete surgical excision and clear margins forming the basis of initial management. Although early postoperative and oncologic outcomes have been favorable, interpretation is limited by the short duration of follow-up and the pending molecular confirmation of the EWSR1-FLI1 translocation. Continued surveillance and completion of genetic studies will be essential to fully characterize disease behavior and guide long-term management.
